# Association between Pancreatic Atrophy and Loss of Insulin Secretory Capacity in Patients with Type 2 Diabetes Mellitus

**DOI:** 10.1155/2019/6371231

**Published:** 2019-08-05

**Authors:** Jun Lu, Meixiang Guo, Hongtao Wang, Haibin Pan, Liang Wang, Xuemei Yu, Xueli Zhang

**Affiliations:** ^1^Department of Endocrinology and Metabolism, Shanghai University of Medicine & Health Sciences Affiliated Fengxian Hospital, 6600 Nanfeng Road, Shanghai 201499, China; ^2^Department of Comprehensive Diagnosis and Treatment for Diabetes, Shanghai University of Medicine & Health Sciences Affiliated Fengxian Hospital, 6600 Nanfeng Road, Shanghai 201499, China; ^3^Department of Radiology, Shanghai University of Medicine & Health Sciences Affiliated Fengxian Hospital, 6600 Nanfeng Road, Shanghai 201499, China; ^4^Department of Biostatistics and Epidemiology, College of Public Health, East Tennessee State University, Johnson City, Tennessee 37614, USA

## Abstract

**Aims:**

To examine pancreatic volume (PV) changes among patients with different duration of type 2 diabetes and whether pancreatic atrophy was associated with loss of insulin secretory capacity.

**Methods:**

This cross-sectional study (203 patients with type 2 diabetes, 93 controls without diabetes) was conducted from January 2016 to December 2017. Patients with type 2 diabetes were divided into 3 groups: recently diagnosed (duration ≤ 2 years), midterm (duration 3-9 years), and long term (duration ≥ 10 years). All the patients were scanned with upper abdominal computerized tomography; PV was then calculated by an experienced technician. Absolute insulin deficiency was defined as fasting C − peptide < 0.9 ng/mL.

**Results:**

Compared with PV (cm^3^) in the controls, the mean PV was similar in patients with recently diagnosed type 2 diabetes (68.8 versus 71.0, *P* = 0.56) but significantly reduced in patients with midterm (68.8 versus 60.8, *P* < 0.05) and long-term (68.8 versus 53.1, *P* < 0.001) type 2 diabetes. A similar trend was observed for the PV index (PV adjusted for body surface area and body mass index). Furthermore, rates of pancreatic atrophy and absolute insulin deficiency increased with duration of diabetes. Multiple logistic regression analysis indicated that pancreatic atrophy was associated with higher likelihood of absolute insulin deficiency (odds ratio = 4.47, 95%confidence interval = 1.45‐13.8).

**Conclusions:**

PV was reduced in those with midterm and long-term type 2 diabetes compared to individuals without type 2 diabetes. Overall, pancreatic atrophy was associated with the loss of insulin secretory capacity in patients with type 2 diabetes.

## 1. Introduction

Morphology of the pancreas has been investigated using ultrasonography, helical computerized tomography (CT), and magnetic resonance imaging [1–5]. In the general population, pancreas volume increases linearly with age during childhood and adolescence, reaches a plateau from age 20 to 60 years, and then declines thereafter [6]. Several pathological conditions (e.g., chronic pancreatitis, diabetes, and carcinoma) affected pancreatic morphology [5–9]. Pancreatic morphology in patients with diabetes has been widely studied. It has been reported that pancreatic volume (PV) was reduced in patients with type 1 diabetes, latent autoimmune diabetes in adults (LADA), or some specific types of diabetes [10–16]. However, pancreatic morphology is a controversial feature in patients with type 2 diabetes [15–19], with several studies reporting a reduced PV in patients with type 2 diabetes [17–19], while other studies reported no difference compared with the normoglycemic controls. Many factors could have accounted for different conclusions, such as body mass index (BMI), body surface area (BSA), abdominal obesity, and body weight, which were strongly related to PV in patients with type 2 diabetes [11, 14, 15]. Recently, researchers pointed out a possible association between PV and duration of type 2 diabetes [17], and more studies are warranted to explore this issue.

Most pancreatic capacity is occupied by exocrine pancreatic acini [20, 21]. However, studies have demonstrated that PV was positively associated with pancreatic *β* cell function in diabetic patients [11, 19, 22]. Moreover, progressive loss of insulin secretory function is an inevitable result of the natural history of type 2 diabetes [23]. It is unclear whether pancreatic atrophy is accompanied by loss of insulin secretory function in patients with long-standing diabetes.

Our previous study indicated that patients with type 2 diabetes had lower PV than the control group [11]. In this study, we expanded the sample size, further investigated the association of PV with duration of diabetes, and explored the relationship between PV feature and loss of insulin secretory capacity in patients with various durations of type 2 diabetes.

### 1.1. Subjects

This study was performed at the Shanghai University of Medicine & Health Sciences Affiliated Fengxian Hospital from January 2016 to December 2017. Patients aged 18–75 years were included in this study. Patients with type 2 diabetes (*n* = 210) were recruited from the inpatient Department of Endocrinology and Metabolism of the Shanghai University of Medicine & Health Sciences Affiliated Fengxian Hospital. Individuals without diabetes (*n* = 100) were recruited from the Department of Surgery. Those with a history of acute or chronic pancreatitis (*n* = 4), cancer (*n* = 3), severe hepatic or renal dysfunction (alanine transaminase ≥ 90 u/L, serum creatinine ≥ 450 *μ*mol/L; *n* = 12), or an unclear image (*n* = 8) were excluded. Finally, 203 diabetic patients and 93 controls were included in this study. Demographic data were collected from patients' records. All the biochemical parameters were assayed in the laboratory of the Shanghai University of Medicine & Health Sciences Affiliated Fengxian Hospital. CT scans were performed in the Department of Radiology in the Shanghai University of Medicine & Health Sciences Affiliated Fengxian Hospital. According to the duration of diabetes, patients were categorized as follows: recently diagnosed (diabetes duration ≤ 2 years) (*n* = 53), midterm (duration 3‐9 years) (*n* = 65), and long term (duration ≥ 10 years) (*n* = 85).

All procedures followed were in accordance with the ethical standards of the responsible committee on human experimentation (institutional and national) and with the Helsinki Declaration of 1975, as revised in 2008. Informed consent was obtained from all patients for being included in the study.

## 2. Materials and Methods

### 2.1. Anthropometric and Biochemical Measurements

Height and weight were measured using standard methods. Height was measured to the nearest 0.1 centimeter, and weight was measured to the nearest 0.1 kilogram. BMI (kg/m^2^) was calculated as weight divided by height squared. BSA (m^2^) was calculated as 0.0061 × height (cm) + 0.0128 × weight (kg) − 0.1529.

Venous blood samples were drawn after an overnight fast (10–16 h). Glycosylated hemoglobin (HbA1c) was determined using high-performance liquid chromatography (HLC-73G7; Tosoh, Tokyo, Japan). Fasting C-peptide (FCP) was measured using electrochemical luminescence, and plasma glucose was measured using the glucose oxidase method (Roche Diagnostics GmbH, Mannheim, Germany). Lipid profiles were measured using an autoanalyzer (Hitachi 7600; Hitachi, Tokyo, Japan). Absolute insulin deficiency was defined as the level of FCP < 0.9 ng/mL.

### 2.2. PV Assessment

All the subjects underwent upper abdominal CT scans with the method describe previously [11]. Scan images were coded and analyzed by an experienced technician; the outline of the region of interest was annotated by freehand using a Siemens Virtuoso workstation, generating a pancreatic area for each slice. The PV was calculated by the sum of the segmental area multiplied by the interval between each slice (5 mm). PV indices were calculated as PV divided by BSA (PVI-BSA) or PV divided by BMI (PVI-BMI). The staff was blinded to the clinical information of the patients when the images were analyzed. Pancreatic atrophy was defined as a PV ≤ 5^th^ percentile of the PV in the control.

### 2.3. Statistical Analysis

The Kolmogorov–Smirnov test was used to assess the distribution of continuous variables. Normally distributed continuous variables were expressed as the mean ± standard deviation, abnormally distributed variables as the median (interquartile range), and categorical variables as the number (percentage). Differences in medians were examined using the Kruskal–Wallis test among three or four groups. We conducted an analysis of a general linear model to draw an overall *P* value of comparison of means (PV, PVI-BSA, and PVI-BMI) among four groups, and between-group comparison was subsequently performed using the LSD method. Differences in proportions were tested using the chi-square test. Multiple logistic regression was used to examine the association between pancreatic atrophy and absolute insulin deficiency after adjusting for sex, age, duration of diabetes, BMI, HbA1c, total cholesterol, and triglyceride. Statistical analyses were performed using SPSS 19.0 (IBM SPSS Statistics for Windows, Version 19.0.; IBM Corp., Armonk, NY); two-sided *P* values < 0.05 were considered statistically significant.

## 3. Results

### 3.1. Clinical Characteristics of the Patients

As shown in Table 1, patients with type 2 diabetes were older and more obese and had higher levels of systolic blood pressure (SBP), diastolic blood pressure (DBP), fasting plasma glucose, HbA1c, and rate of hypertension than the control group (*P* < 0.05). There were no significant differences in levels of total cholesterol, triglyceride, high-density lipoprotein-cholesterol (HDL-C), low-density lipoprotein-cholesterol (LDL-C), BSA, serum creatinine, and alanine transaminase (ALT) among the groups.

### 3.2. Comparison of PV among Patients with Type 2 Diabetes and Controls

There were significant differences in the mean PV (cm^3^) among the four groups (*P* < 0.001, Table 2). The corresponding mean PVs were 68.8 cm^3^ (controls), 71.0 cm^3^ (recently diagnosed), 60.3 cm^3^ (midterm), and 53.1 cm^3^ (long term). The recently diagnosed diabetic patients had similar PV to the controls. However, midterm and long-term diabetic patients had significantly lower PV (cm^3^) than the controls (60.8 vs. 68.8, *P* < 0.05; 53.1 vs. 68.8, *P* < 0.001, respectively) and recently diagnosed diabetic patients (60.8 vs. 71.0, *P* < 0.05; 53.1 vs. 71.0, *P* < 0.001, respectively). There were also significant differences in PV (cm^3^) between midterm and long-term diabetic patients (60.8 vs. 53.1, respectively, *P* < 0.05). Additionally, patients with long-term diabetes had lower PVI-BSA and PVI-BMI compared with the controls and recently diagnosed diabetic individuals (all *P* < 0.05).

### 3.3. Association between Pancreatic Atrophy and Absolute Insulin Deficiency

**Figure 1:**
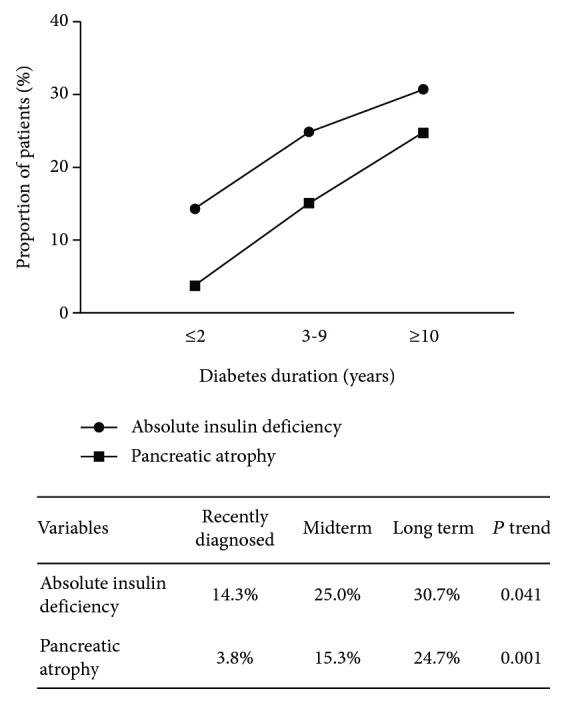
Proportion of absolute insulin deficiency (FCP< 0.9 ng/mL) and pancreatic atrophy in patients with various durations of type 2 diabetes.

The rate of pancreatic atrophy increased sharply with duration, from 3.8% in recently diagnosed diabetes, 15.3% in midterm diabetes, to 24.7% in long-term diabetes (*P* for trend < 0.01, Figure 1). Meanwhile, the proportion of patients with absolute insulin deficiency also escalated along with duration of diabetes (*P* for trend < 0.05). As shown in Table 3, multiple logistic regression analysis showed that pancreatic atrophy was associated with higher likelihood of having absolute insulin deficiency (odds ratio = 4.47, 95%confidence interval = 1.45‐13.8). Moreover, PVI-BSA and PVI-BMI were closely associated with absolute insulin deficiency (*r* ≈ 0.4, *P* < 0.001).

## 4. Discussion

To our knowledge, this is among the first studies to examine the association between pancreatic atrophy and absolute insulin deficiency among a Chinese diabetic population. Our major findings are (1) patients with recently diagnosed type 2 diabetes had comparable PV to the controls, (2) PV was reduced in patients with midterm and long-term type 2 diabetes, and (3) pancreatic atrophy was associated with absolute insulin deficiency.

Previous findings about the pancreatic morphology in patients with type 2 diabetes were controversial. There were three studies which reported reduced PV in patients with type 2 diabetes [17–19]. Our previous study also reported a reduced PV in type 2 diabetes [11]. However, the relationship between PV and duration of diabetes has not been fully investigated in patients with type 2 diabetes. One study reported that the PV decreased in patients with duration ≥ 5 years compared to those with duration under 5 years [17], but PV in patients with long-term diabetes has not been investigated. This study showed that PV decreased in patients with mid- and long-term type 2 diabetes and that pancreatic atrophy was more frequent in patients with long-term type 2 diabetes. Additionally, patients with recently diagnosed type 2 diabetes had comparable PV to the normoglycemic controls. Therefore, difference in duration may partially account for the inconsistent conclusions of PV features in patients with type 2 diabetes from previous studies.

Previous studies indicated that PV was positively associated with *β* cell function [11, 12, 16, 24, 25]; this study showed that pancreatic atrophy was positively associated with absolute insulin deficiency. The mechanism of the correlation between PV and *β* cell secretory function was complicated. On the one hand, pancreatic volume parameters are postulated to represent the number of islets and insulin secretory capacity [12, 24, 25]. Pancreatic islets could secrete enough insulin to maintain the blood glucose level in rat after 50% pancreatectomy [19]. In humans, patients who underwent more than 80% of pancreatectomy have a high incidence of diabetes (66.7%) and all the patients who underwent over 90% of pancreatectomy became diabetic [12]. On the other hand, the insulin secretory capacity of the islets is considered very important to maintain PV. The beta cell regulated growth and amylase synthesis of pancreatic acinar cells via the islet-acinar axis [26]. A recent study demonstrated that a significant change in the irregularity of the pancreatic borders occurred after restoration of normal insulin secretion [27].

There were some limitations in this study. First, this is a cross-sectional study. Second, exocrine secretory function of the pancreas has not been determined, which has been postulated to decrease in patients with long-term type 2 diabetes. Third, the sample may not be large enough for examining the association between pancreatic atrophy and absolute insulin deficiency according to duration of type 2 diabetes. However, the 95% CI is still wide for the odds of pancreatic atrophy associated with absolute insulin deficiency in the overall sample of diabetes patients (OR = 4.47, 95%CI = 1.45‐13.8).

In conclusion, PV was reduced in patients with midterm and long-term type 2 diabetes, but not in recently diagnosed diabetic individuals. Pancreatic atrophy was associated with the loss of insulin secretory capacity in patients with type 2 diabetes. Longitudinal studies with large sample size are needed to further study the association between pancreatic atrophy and absolute insulin deficiency.

## Figures and Tables

**Table 1:** Characteristics of participants.

Variables	Controls (*n* = 93)	Patients with type 2 diabetes				*P* value
		Overall (*n* = 203)	≤2 years (*n* = 53)	3~9 years (*n* = 65)	≥10 years (*n* = 85)	
Gender (men)	49 (52.7)	123 (60.6)	31 (58.5)	42 (63.6)	50 (58.8)	0.31
Age (years)	47 (35-59)	60 (53-65)^‡^	59 (52-65)	57 (51-65)	63 (58-66)	<0.001
Duration of diabetes (years)^∗^	ND	8 (2-12)	0.7 (0.1-1)	6 (5-8)	13 (11-18)	<0.001
BSA (m^2^)	1.66 ± 0.18	1.71 ± 0.19^†^	1.69 ± 0.19	1.72 ± 0.20	1.70 ± 0.19	0.16
BMI (kg/m^2^)	22.7 ± 3.11	24.4 ± 3.68^‡^	25.1 ± 3.18	23.9 ± 3.23	24.5 ± 4.25	<0.001
HTN	21 (22.6)	76 (37.4)^†^	15 (28.3)	23 (35.4)	38 (44.7)	0.014
SBP (mmHg)	120 (115-127)	130 (120-140)^‡^	130 (120-135)	126 (117-134)	130 (120-145)	<0.001
DBP (mmHg)	75 (70-80)	80 (70-84)^†^	80 (75-84)	79 (70-80)	80 (70-85)	0.001
FCP (ng/mL)^∗^	ND	1.80 ± 1.27	1.87 ± 0.97	1.76 ± 1.17	1.78 ± 1.49	0.90
FPG (mmol/L)	5.09 (4.76-5.41)	7.61 (6.32-10.1)^‡^	7.14 (6.40-8.25)	7.92 (6.16-11.3)	7.97 (6.68-10.7)	<0.001
HbA1c (%)	5.8 (5.3-6.1)	7.8 (6.7-9.5)^†^	6.9 (6.1-9.8)	7.5 (6.5-9.3)	8.5 (7.2-9.7)	0.003
ALT (U/L)	18 (11-33)	19 (13-26)	21 (16-27)	18 (13-25)	17 (13-26)	0.05
TG (mmol/L)	1.21 (1.01-1.55)	1.36 (0.89-2.06)	1.52 (1.18-2.36)	1.25 (0.76-1.67)	1.31 (0.83-2.10)	0.39
TC (mmol/L)	4.57 ± 0.95	4.76 (3.94-5.5)	4.71 ± 1.00	4.70 ± 1.02	4.80 ± 1.17	0.85
HDL-C (mmol/L)	1.10 (0.96-1.23)	1.12 (0.96-1.35)	1.12 (0.96-1.37)	1.17 (1.00-1.34)	1.10 (0.93-1.32)	0.77
LDL-C (mmol/L)	3.03 ± 0.79	2.68 (2.15-3.37)	2.65 ± 0.81	2.77 ± 0.82	2.88 ± 0.98	0.33
SCR (*μ*mol/L)	65 (57-79)	65 (53-76)	65 (52-71)	67 (52-77)	67 (54-81)	0.80
Insulin deficiency (FCP < 0.9 ng/nL)^∗^	ND	44 (21.7)	7 (14.3)	14 (21.5)	23 (30.7)	0.038

^∗^ *P* values were analyzed among the diabetes subgroups. ^†^*P* < 0.05 versus the controls; ^‡^*P* < 0.001 versus the controls. Data are presented as the median (interquartile range) or mean ± standard deviation for continuous variables and the number (percentage) for category variables. Differences in medians/means were examined using the Kruskal–Wallis test or a general linear model among three or four groups; differences in proportions were tested using the chi-square test. Abbreviations: BMI: body mass index; BSA: body surface area; DBP: diastolic blood pressure; FCP: fasting C-peptide; FPG: fasting plasma glucose; HDL-C: high-density lipoprotein-cholesterol; HTN: hypertension; LDL-C: low-density lipoprotein-cholesterol; ND: not determined; SBP: systolic blood pressure; TC: total cholesterol; TG: triglyceride.

**Table 2:** Differences in PV, PVI-BSA, and PVI-BMI in various subgroups.

Variables	Control	Patients with type 2 diabetes				*P* for trend
		Overall	≤2 years	3~9 years	≥10 years	
PV (cm^3^)	68.8 ± 20.3	60.2 ± 23.3^†^	71.0 ± 22.6	60.8 ± 21.8^†#^	53.1 ± 22.5^∗^^‡※^	<0.001
PVI-BSA (cm^3^/m^2^)	41.4 ± 11.0	35.2 ± 12.6^∗^	41.7 ± 12.0	35.2 ± 11.2^†#^	30.8 ± 12.4^∗^^‡※^	<0.001
PVI-BMI (cm^3^/kg/m^2^)	3.06 ± 0.91	2.48 ± 0.92^∗^	2.83 ± 0.88	2.56 ± 0.84^†^	2.17 ± 0.92^∗^^‡※^	<0.001

PVI-BSA: PV adjusted for body surface area; PVI-BMI: PV adjusted for body mass index. ^∗^*P* < 0.001 and ^†^*P* < 0.05 compared to the control. ^‡^*P* < 0.001 and ^#^*P* < 0.05 compared to patients with duration ≤ 2 years. ^※^*P* < 0.05 compared to patients with duration 3-9 years. *P* for trend was calculated using a general linear model.

**Table 3:** The association of pancreatic atrophy and absolute insulin deficiency (FCP < 0.9 ng/mL) in patients with type 2 diabetes.

Variables	OR (95% CI)	*P* value
Pancreatic atrophy (%)	4.47 (1.45-13.8)	0.009
Gender (men)	0.53 (0.21-1.33)	0.18
Age (years)	0.98 (0.94-1.02)	0.37
Duration of diabetes^∗^	1.03 (0.96-1.10)	0.39
BMI (kg/m^2^)	0.88 (0.77-0.99)	0.047
HbA1c (%)	1.26 (1.06-1.48)	0.008
TG (mmol/L)	0.77 (0.54-1.08)	0.13
TC (mmol/L)	1.49 (0.95-2.33)	0.08

^∗^Duration of diabetes was categorized as ≤2 years, 3-9 years, and ≥10 years. Abbreviations: BMI: body mass index; HTN: hypertension; TC: total cholesterol; TG: triglyceride; OR: odds ratio; CI: confidence index.

## Data Availability

The data are available under the consent of the corresponding author.

## Abbreviations

CT: Computed tomography
FCP: Fasting C-peptide
FPG: Fasting plasma glucose
BMI: Body mass index
BSA: Body surface area
TC: Total cholesterol
TG: Triglycerides
HDL-C: High-density lipoprotein-cholesterol
LDL-C: Low-density lipoprotein-cholesterol
SBP: Systolic blood pressure
DBP: Diastolic blood pressure
PV: Pancreatic volume
PVI-BMI: Pancreatic volume index adjusted for BMI
PVI-BSA: Pancreatic volume index adjusted for BSA.
